# Cost-effectiveness of osimertinib versus standard EGFR-TKI as first-line treatment for EGFR-mutated advanced non-small-cell lung cancer in China

**DOI:** 10.3389/fphar.2022.920479

**Published:** 2022-09-20

**Authors:** Yamin Shu, Yufeng Ding, Xucheng He, Yanxin Liu, Pan Wu, Qilin Zhang

**Affiliations:** ^1^ Department of Pharmacy, Tongji Hospital, Tongji Medical College, Huazhong University of Science and Technology, Wuhan, China; ^2^ Department of Pharmacy, Pengzhou Second People’s Hospital, Pengzhou, China; ^3^ Department of Pharmacy, Pengzhou People’s Hospital, Pengzhou, China; ^4^ Department of Pharmacy, Qionglai Maternal and Child Health and Family Planning Service Center, Qionglai, China; ^5^ Department of Pharmacy, Union Hospital, Tongji Medical College, Huazhong University of Science and Technology, Wuhan, China

**Keywords:** cost-effectiveness analysis, first-line treatment, FLAURA trial, non-small-cell lung cancer, osimertinib

## Abstract

**Objective:** The purpose of this study was to estimate the cost-effectiveness of osimertinib for the first-line treatment of patients with EGFR-mutated advanced non-small-cell lung cancer (NSCLC) from the perspective of the Chinese healthcare system.

**Methods:** A Markov model was developed to simulate the outcomes and direct medical costs of osimertinib or standard EGFR-TKI in the first-line treatment of patients with previously untreated EGFR-mutated advanced NSCLC. Individual patient survival data were extracted from the FLAURA randomized clinical trial. Clinical costs and utilities’ input estimates were collected from the local hospital and available literature reports. The quality-adjusted life-year (QALY), incremental cost-effectiveness ratio (ICER), incremental net monetary benefit (INMB), and incremental net health benefit (INHB) were calculated for the two treatment strategies over a 10-year lifetime horizon. In addition, one-way sensitivity analysis, probabilistic sensitivity analysis, and subgroup analysis were performed to test the robustness of the model.

**Results:** On baseline analysis, osimertinib achieved additional 0.39 QALYs and $15,443.78 incremental costs compared with standard EGFR-TKI (gefitinib or erlotinib), which resulted in the ICER of $39,369.53/QALY. The INMB was -$755.11, and the INHB was -0.02 QALYs at a WTP threshold of $37,663.26/QALY in China. The one-way sensitivity analysis showed that the utility of PFS had the strongest association with the ICER. Osimertinib had approximately 46.4% probability of being cost-effective at the WTP threshold of $37,663.26/QALY.

**Conclusion:** First-line osimertinib therapy might not be cost-effective in China for patients with EGFR-mutated advanced NSCLC compared with standard EGFR-TKI based on its current marketed price. A significantly more favorable cost-effectiveness could be achieved when the price of osimertinib was reduced by 5%.

## Introduction

Lung cancer is the second most common malignant cancer and the leading cause of cancer-related deaths in the world, with a 5-year survival rate of less than 20% (Siegel et al., 2021; Sung et al., 2021). In China, lung cancer ranks first in both morbidity and mortality of all malignant cancers (Chen et al., 2016). There are two main histopathological types of lung cancer: non-small-cell lung cancer (NSCLC) and small-cell lung cancer; of these, NSCLC is the most common subtype, accounting for 85–90% of all lung cancers (Reck and Rabe, 2017). Epidermal growth factor receptor (EGFR) sensitive mutations play an important role in NSCLC disease progression. In China, about 35–40% of NSCLC in patients are caused by EGFR-coding gene mutation (Hsu et al., 2018).

Currently, EGFR tyrosine kinase inhibitors (EGFR-TKIs) have been recommended by many guidelines as the standard first-line therapy for patients with EGFR mutation-positive advanced NSCLC (Wang et al., 2021; Ettinger et al., 2022). Studies have shown that treatment of the first- and second-generation of EGFR-TKIs, including gefitinib, erlotinib, and afatinib, could significantly prolong the progression-free survival (PFS) and improve the response rate of lung cancer patients compared with the standard chemotherapy as initial therapy (Mitsudomi et al., 2010; Wu et al., 2015; Yang et al., 2015). In a meta-analysis of six randomized trials involving NSCLC patients who had not previously received treatment, the median PFS for patients using EGFR-TKI (gefitinib or erlotinib) was 11.0 months, compared with only 5.6 months in the chemotherapy group (Lee et al., 2017). However, the vast majority of patients eventually developed disease progression after 9–13 months, of which approximately 60% were resistant due to the T790M mutation (Morgillo et al., 2016; Mok et al., 2017).

Osimertinib is a new oral, irreversible, third-generation EGFR-TKI that is designed to selectively target inhibition of both EGFR sensitizing and T790M resistance mutations and also has therapeutic effects on NSCLC central nervous system (CNS) metastasis (Mok et al., 2017; Yang et al., 2017; Goss et al., 2018). Osimertinib received accelerated approval by the US Food and Drug Administration (FDA) in November 2015 and was conventionally approved in March 2017 for the treatment of metastatic EGFR T790M mutation-positive NSCLC in patients who have disease progression during or after EGFR-TKI therapy, which was currently the first effective marketed drug targeting the T790M resistance mutation (Osimertinib (TAGRISSO), 2017). In NSCLC patients with EGFR mutations (exon 19 deletion or L858R allele), the clinical practice guidelines of the 2022 National Comprehensive Cancer Network (NCCN) give priority to osimertinib (Ettinger et al., 2022). FLAURA was a randomized, double-blind phase 3 trial designed to explore the efficacy and safety of osimertinib versus first-generation EGFR-TKI (gefitinib or erlotinib) in the first-line therapy for patients with EGFR mutation-positive advanced NSCLC. The results showed that the median PFS (18.9 vs. 10.2 months, HR = 0.46, 95% CI: 0.37 to 0.57, *p* < 0.001) and the overall survival (38.6 vs. 31.8 months, HR = 0.80, 95.05% CI: 0.64 to 1.00, *p* = 0.046) of osimertinib were demonstrated to be significantly longer than standard EGFR-TKI, with a similar safety profile and low rates of serious adverse events (SAEs) (Soria et al., 2018; Ramalingam et al., 2020).

Osimertinib was approved for marketing in China in 2017 due to its remarkable clinical benefit and safety in NSCLC population. However, the price of osimertinib in China is higher than that of gefitinib and erlotinib (Cai et al., 2019). Moreover, the economic evaluation of osimertinib treatment in China is limited. Therefore, the purpose of this study was to evaluate the economics of osimertinib versus first-generation EGFR-TKI (gefitinib or erlotinib) as the first-line treatment of untreated EGFR mutation-positive advanced NSCLC based on the FLAURA trial results from the perspective of the Chinese healthcare system, in order to provide reference for clinical treatment option and medical decision-making in China.

## Methods

### Analytical overview and model structure

**FIGURE 1:**
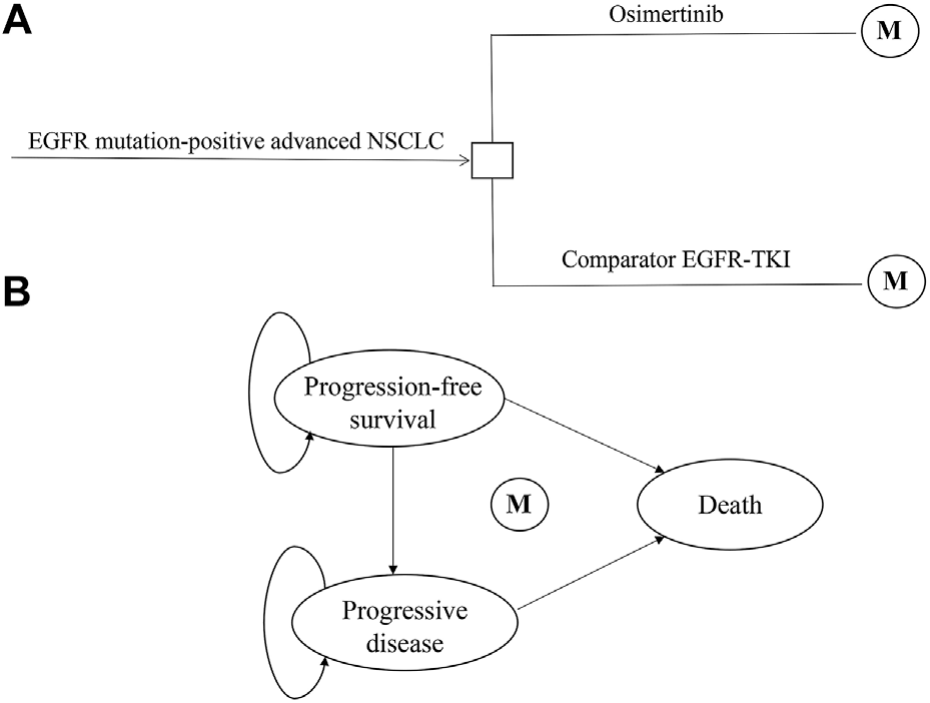
Model structure of a decision tree combining the Markov state transition model with the three health states. **(A)** Decision tree. **(B)** Markov state transition model. Abbreviations: M, Markov node; EGFR-TKI, epidermal growth factor receptor–tyrosine kinase inhibitor; NSCLC, non-small-cell lung cancer.

The hypothetical target population in our study was consistent with the characteristics of patients in the FLAURA trial. Patients with advanced or metastatic NSCLC, who had not been treated previously, were eligible to receive first-line treatment with osimertinib, gefitinib, or erlotinib. A local or central test confirmed the EGFR-TKI sensitivity, exon 19 deletion (Ex19del), or p. Leu858Arg (L858R) EGFR mutation, alone or in combination with other EGFR mutations. Patients with CNS metastases whose condition was neurologically stable were eligible. Complete eligibility criteria were available in the FLAURA trial protocol (Soria et al., 2018; Ramalingam et al., 2020). A decision tree and three health-state Markov model were constructed by TreeAge Pro 2019 to estimate clinical and economic outcomes of osimertinib versus comparator EGFR-TKI as the first-line treatments for patients with untreated EGFR-mutated advanced NSCLC in China (Figure 1). The three mutually exclusive health states included PFS, progressive disease (PD), and death. The survival ratios with PFS and PD were estimated from the area under the PFS and OS Kaplan–Meier survival curves in the FLAURA trial. The simulation time horizon was 10 years, with a cycle length of 1 month, and a half-cycle correction was adopted to adjust the head–tail bias of cycles in the model. The subsequent annual costs and benefits were reduced using a discount rate of 3%, according to the WHO guidelines for pharmacoeconomic evaluations (Murray et al., 2000).

All costs had been adjusted to 2022 prices according to the local Consumer Price Index and were presented in US dollars ($1 = ¥6.45, 2021). The willingness-to-pay (WTP) threshold used 3× the per capita gross domestic product (GDP) in 2021 ($37,663.26/per additional QALY gained) of China (National Bureau of Statistics of China, 2022) because of the developing country. All economic evaluation and survival analyses were conducted by TreeAge Pro software (version 2019, https://www.treeage.com/) and R software (version 4.0.5, http://www.r-project.org). This study did not need approval from an institutional review board or ethics committee because it was based on publicly available clinical trial data and modeling techniques.

### Clinical data

**FIGURE 2:**
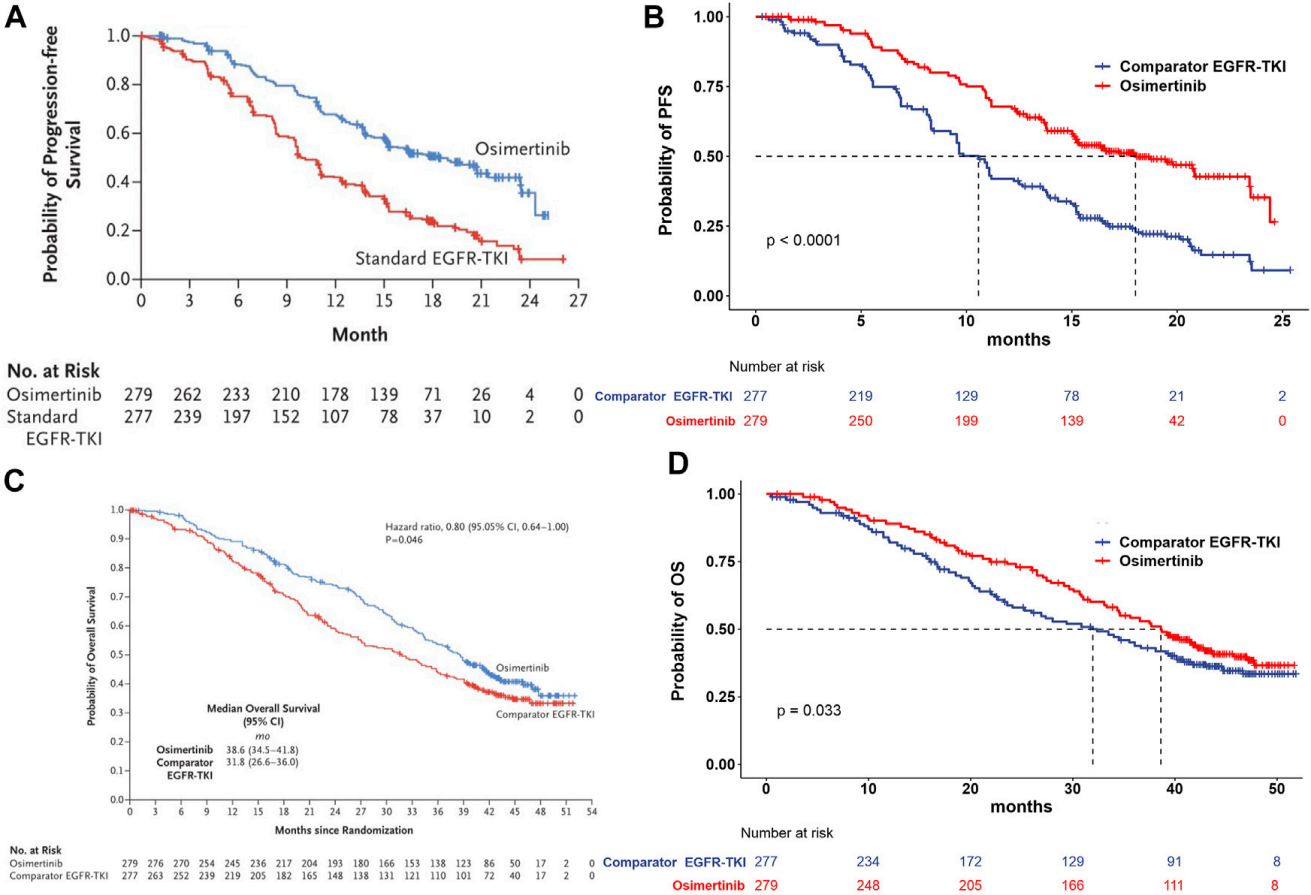
Model estimated PFS and OS were plotted together with the original Kaplan–Meier PFS and OS curves from the FLAURA trial, respectively. **(A)** Kaplan–Meier curve of the progression-free survival from the FLAURA trial. **(B)** Simulation of the progression-free survival curve for osimertinib and the comparator EGFR-TKI. **(C)** Kaplan–Meier curve of overall survival from the FLAURA trial. **(D)** Simulation of the overall survival curve for osimertinib and the comparator EGFR-TKI. Abbreviations: PFS, progression-free survival; OS, overall survival; EGFR-TKI, epidermal growth factor receptor–tyrosine kinase inhibitor.

The treatment groups in our study were included from the FLAURA trial. The eligible patients were randomly assigned in a 1:1 ratio to receive either osimertinib (n = 279, at a dose of 80 mg once daily) or a standard EGFR-TKI (n = 277, erlotinib at a dose of 150 mg once daily or gefitinib at a dose of 250 mg once daily) (Soria et al., 2018; Ramalingam et al., 2020). The treatment continued until disease progression or the development of unacceptable toxicity. The median duration of total treatment exposure was 16.2 months (0.1–27.4) for patients receiving osimertinib and 11.5 months (0–26.2) for those receiving a standard EGFR-TKI. Transition probabilities between different health states were estimated from Kaplan–Meier survival curves which were obtained from the FLAURA trial, and the curves beyond the model time horizon were extrapolated by standard statistical analyses. GetData Graph Digitizer software (Version 2.26, http://getdata-graph-digitizer.com/index.php) was used to digitize data points of Kaplan–Meier of PFS and OS curves in the full analysis set, due to the unavailable individual survival data in trial. The valid data points were used to simulate the survival curves by R software, and then, survival distributions (Weibull, log-logistic, log-normal, gamma, and exponential distributions) were used to extrapolate the probability of survival beyond the observation period, and the corresponding parameters were obtained simultaneously. The selection criteria for optimal parameter survival distribution were based on the minimum value of the Akaike information criterion (AIC) (Guyot et al., 2012). The final survival curve simulation and relevant parameters are shown in Figure 2 and Table 1.

**TABLE 1:** Relevant parameters of different survival distribution.

Parameter	Value
PFS of treatment with osimertinib	
Weibull	Shape = 1.676, scale = 0.005, and AIC = 1136.085
Log-logistic	Shape = 2.017, scale = 17.692, and AIC = 1130.980
Log-normal	Meanlog = 2.886, sdlog = 0.870, and AIC = 1128.339
Gamma	Shape = 2.169, rate = 0.104, and AIC = 1132.214
Exponential	Rate = 0.036 and AIC = 1172.031
PFS of treatment with comparator EGFR-TKI	
Weibull	Shape = 1.502, scale = 0.019, and AIC = 1452.477
Log-logistic	Shape = 1.990, scale = 10.299, and AIC = 1454.498
Log-normal	Meanlog = 2.304, sdlog = 0.908, and AIC = 1463.449
Gamma	Shape = 1.875, rate = 0.146, and AIC = 1451.190
Exponential	Rate = 0.071 and AIC = 1491.060
OS of treatment with osimertinib	
Weibull	Shape = 1.664, scale = 0.002, and AIC = 1517.047
Log-logistic	Shape = 1.986, scale = 38.309, and AIC = 1518.256
Log-normal	Meanlog = 3.643, sdlog = 0.898, and AIC = 1519.491
Gamma	Shape = 2.071, rate = 0.046, and AIC = 1516.333
Exponential	Rate = 0.017 and AIC = 1556.495
OS of treatment with comparator EGFR-TKI	
Weibull	Shape = 1.290, scale = 0.008, and AIC = 1586.764
Log-logistic	Shape = 1.602, scale = 31.409, and AIC = 1585.105
Log-normal	Meanlog = 3.455, sdlog = 1.162, and AIC = 1598.021
Gamma	Shape = 1.445, rate = 0.035, and AIC = 1586.019
Exponential	Rate = 0.022 and AIC = 1597.351

PFS, progression-free survival; OS, overall survival; AIC, Akaike information criterion; EGFR-TKI, epidermal growth factor receptor–tyrosine kinase inhibitor.

### Cost and utility values

From the perspective of the Chinese healthcare system, all costs in the model were from local medical organizations or previously published literature reports (Zhang et al., 2016; Guan et al., 2019; Wu et al., 2019; Shu et al., 2021). Only direct medical costs, including costs of osimertinib, comparator EGFR-TKI, EGFR mutation testing, management of grade ≥3 SAEs, subsequent therapy, routine follow-up, and terminal care in end-of-life were analyzed in our model (Table 2). After the disease progression, osimertinib or salvage chemotherapy (pemetrexed and cisplatin) would be received as the subsequent active therapy, according to the FLAURA trial and treatment guidelines for NSCLC. To calculate the dosage of chemotherapy, it was assumed that a base-case patient had a height of 1.64 m and a weight of 65 kg, resulting in a body surface area (BSA) of 1.72 m^2^. The most common SAEs extracted from the FLAURA trial, including rash, anemia, alanine aminotransferase (ALT) increased, and aspartate aminotransferase (AST) increased. The costs related to SAEs were calculated through multiplying the incidence of the SAEs by the costs of managing the SAEs per event. Furthermore, mutation testing and terminal cost were also implemented in the first and end cycles, respectively, according to the TreeAge Pro 2019 manual.

**TABLE 2:** Parameter input to the models.

Variable	Base case (range)	Distribution	Source
Costs ($)			
Osimertinib (80 mg)	28.84 (23.07–34.61)	Triangle	Local hospital
Gefitinib (250 mg)	24.74 (19.79–29.69)	Triangle	Local hospital
Erlotinib (150 mg)	12.56 (10.05–15.07)	Triangle	Local hospital
Pemetrexed (200 mg)	201.55 (161.24–241.86)	Triangle	Local hospital
Cisplatin (10 mg)	6.17 (4.94–7.40)	Triangle	Local hospital
Cost of EGFR mutation testing	441 (352.80–529.20)	Triangle	Wu et al. (2019)
Routine follow-up cost per cycle	178.57 (142.86–214.28)	Triangle	Shu et al. (2021)
Cost of terminal care in end-of-life	2583.37 (2066.70–3100.04)	Triangle	Shu et al. (2021)
Costs of SAE per unit ($)			
Rash	5.50 (4.40–6.60)	Triangle	Zhang et al. (2016)
Anemia	614 (491.20–736.80)	Triangle	Guan et al. (2019)
ALT/AST increased	216.35 (173.08–259.62)	Triangle	Zhang et al. (2016)
Risks of serious adverse events in the osimertinib group (grade≥3) %			
Rash	1.08 (0.86–1.30)	Beta	Soria et al. (2018); Ramalingam et al. (2020)
Anemia	2.51 (2.01–3.01)	Beta	Soria et al. (2018); Ramalingam et al. (2020)
ALT increased	0.72 (0.58–0.86)	Beta	Soria et al. (2018); Ramalingam et al. (2020)
AST increased	0.72 (0.58–0.86)	Beta	Soria et al. (2018); Ramalingam et al. (2020)
Risks of serious adverse events in the comparator EGFR-TKI group (grade≥3) %			
Rash	7.22 (5.78–8.66)	Beta	Soria et al. (2018); Ramalingam et al. (2020)
Anemia	1.08 (0.86–1.30)	Beta	Soria et al. (2018); Ramalingam et al. (2020)
ALT increased	7.58 (6.06–9.10)	Beta	Soria et al. (2018); Ramalingam et al. (2020)
AST increased	4.33 (3.46–5.20)	Beta	Soria et al. (2018); Ramalingam et al. (2020)
Utility value			
PFS	0.804 (0.536–0.883)	Beta	Nafees et al. (2017)
PD	0.321 (0.050–0.473)	Beta	Nafees et al. (2017)
Body surface area (m^2^)	1.72 (1.38–2.06)	Triangle	Zhang et al. (2021)
Discount rate (%)	3%	Fixed in PSA	Murray et al. (2000)
HR for overall PFS	0.46 (0.37–0.57)	Beta	Soria et al. (2018)
HR for OS	0.79 (0.63–0.98)	Beta	Ramalingam et al. (2020)
HR for PFS in Asia	0.55 (0.42–0.72)	—	Soria et al. (2018)
HR for PFS in non-Asia	0.34 (0.23–0.48)	—	Soria et al. (2018)
HR for OS in Asia	1.00 (0.75–1.32)	—	Ramalingam et al. (2020)
HR for OS in non-Asia	0.54 (0.38–0.77)	—	Ramalingam et al. (2020)

SAE, serious adverse event; PFS, progression-free survival; PD, progressive disease; PSA, probabilistic sensitivity analysis; ALT, alanine aminotransferase; AST, aspartate aminotransferase; EGFR-TKI, epidermal growth factor receptor tyrosine kinase inhibitor; HR, hazard ratio.

As not reported in the FLAURA trial, the utility values in health states were gained from an international health-related quality of life (HRQL) survey using the EQ-5D instrument (Nafees et al., 2017). The utility values of PFS, PD, and death were 0.804, 0.321, and 0, respectively, which were consistent with an earlier cost-effectiveness analysis evaluating EGFR-TKI for advanced NSCLC in China (Shu et al., 2021).

### Base-case analysis

The main outcomes were cost, quality-adjusted life-years (QALYs), incremental cost-effectiveness ratio (ICER), incremental net monetary benefit (INMB), and incremental net health benefit (INHB) in our analysis. If the ICER was below the prespecified WTP threshold ($37,663.26/QALY) or the INMB/INHB was a positive value, osimertinib would be identified as cost-effective, according to the recommendation. ICER, INMB, and INHB were counted based on the following formulas: ICER=(Co-Cc)/(Eo-Ec) = ΔC/∆E, INMB=(Eo-Ec)*λ-(Co-Cc) = ∆E*λ-ΔC, and INHB= (Eo-Ec)-(Co-Cc)/λ = ∆E-ΔC/λ, where Cx and Ex were the cost and effectiveness of osimertinib (x = o) or the comparator EGFR-TKI (x = c), respectively, and λ was the WTP threshold (Stinnett and Mullahy, 1998; Craig and Black, 2001).

### Sensitivity and subgroup analyses

To explore the influence of input parameters on the model uncertainty, one-way sensitivity analysis and probabilistic sensitivity analysis (PSA) were performed in TreeAge. In the one-way sensitivity analysis, all parameters varied independently of their corresponding lower and upper boundaries, with a range of ±20% or 95% confidence interval (95% CI) of the base-case value so as to confirm the parameters that significantly influenced the ICER. The result of the one-way sensitivity analysis was presented in a Tornado diagram. In PSA, a Monte Carlo simulation with 1000 iterations was performed by simultaneously varied prespecified distributions, and then, a cost-effectiveness acceptability curve and a scatter plot were finally generated to show the possibility that osimertinib would be considered to be cost-effective under various WTP levels.

To evaluate the uncertainty of economic outcomes caused by the race (Asian and non-Asian), additional subgroup analyses were performed in the prespecified subgroup by altering the HRs of PFS and OS in the FLAURA trial. The general cumulative hazard function was H(t) = ‐ln{S(t)} and HR = H(t)_1_/H(t)_2_, where S(t) represents the cumulative survival function, and H(t) represents the cumulative risk function. All parameters with the value range and corresponding distributions are shown in Table 2.

## Results

### Base-case analysis

The base-case analysis showed a time horizon of 10 years. In comparison with the comparator EGFR-TKI, osimertinib obtained an additional 0.39 QALY, with an incremental cost of $15,443.78, resulting in an ICER of $39,369.53/QALY. The INMB was ‐$755.11, and the INHB was ‐0.02 at a Chinese WTP threshold of $37,663.26/QALY (Table 3). The results showed that osimertinib was not a cost-effective treatment strategy compared with the comparator EGFR-TKI in China.

**TABLE 3:** Cost and outcome results in the base-case analysis.

Parameter	Osimertinib group	Comparator EGFR-TKI group
Cost ($)		
PFS state	19,744.61	11,372.29
PD state	29,904.66	22,833.20
Total cost	49,649.27	34,205.49
Incremental cost ($)	15,443.78	—
Effectiveness (QALY)		
PFS state	1.35	0.87
PD state	0.55	0.64
Total effectiveness	1.90	1.51
Incremental effectiveness (QALY)	0.39	—
ICER ($/QALY)	39,369.53	—
INMB ($)	−755.11	—
INHB (QALY)	−0.02	—

PFS, progression-free survival; PD, progressive disease; QALY, quality-adjusted life-year; ICER, incremental cost-effectiveness ratio; EGFR-TKI, epidermal growth factor receptor–tyrosine kinase inhibitor; INMB, incremental net monetary benefit; INHB, incremental net health benefit.

### Sensitivity and subgroup analyses

**FIGURE 3:**
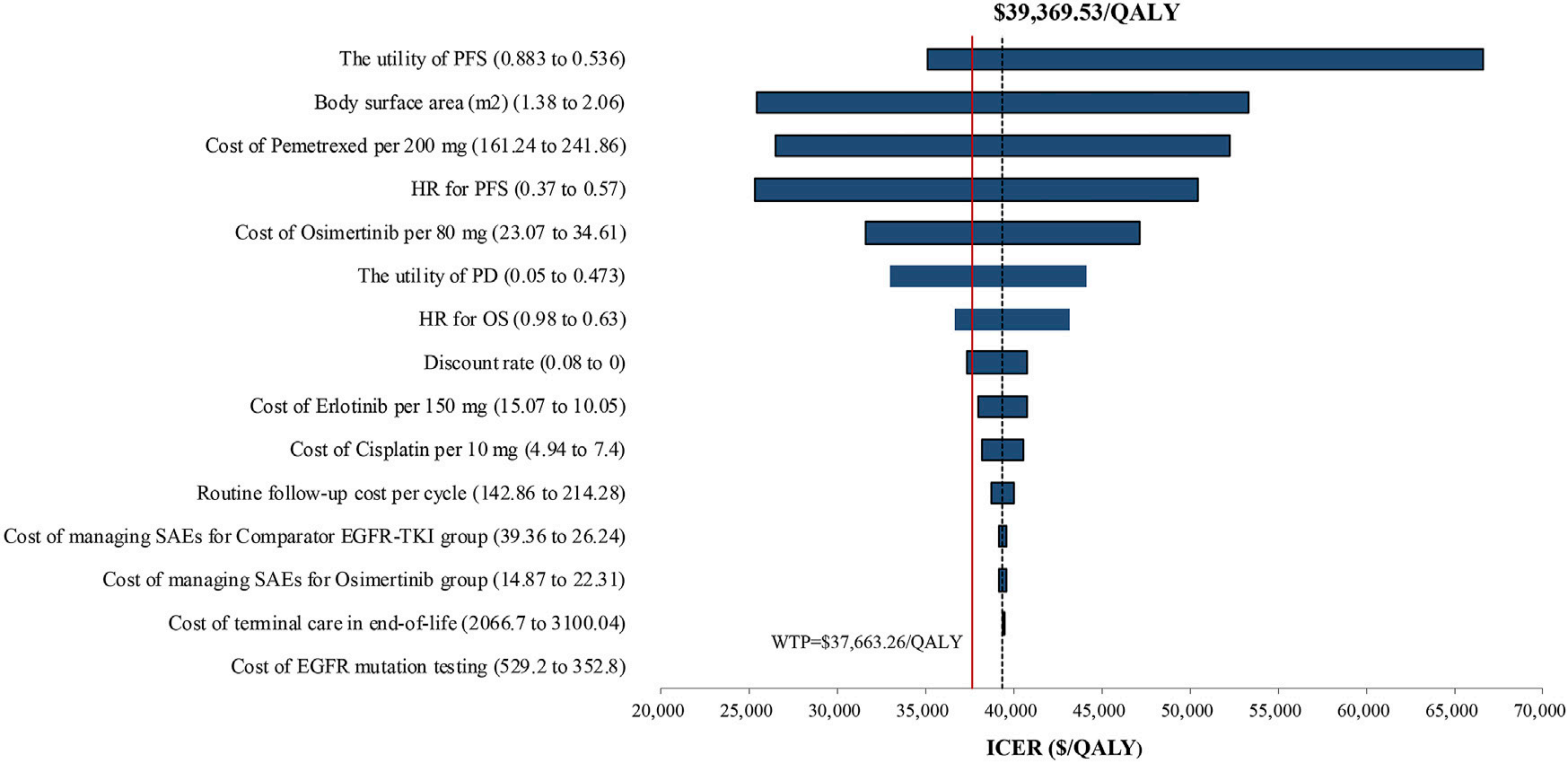
Tornado diagram of one-way sensitivity analysis. It summarized the results of the one-way sensitivity analysis, which listed influential parameters in a descending order according to their effect on the ICER over the variation of each parameter value. Abbreviations: ICER, incremental cost-effectiveness ratio; PFS, progression-free survival; PD, progressive disease; SAEs, serious adverse events; EGFR-TKI, epidermal growth factor receptor–tyrosine kinase inhibitor; HR, hazard ratio.

One-way sensitivity analysis of key variables revealed that the specified variation of the utility of PFS, body surface area, cost of pemetrexed, HR for PFS, cost of osimertinib, the utility of PD, HR for OS, and discount rate might induce the ICER lower than the WTP threshold of $37,663.26/QALY. A Tornado diagram summarizing the results of one-way sensitivity analysis is shown in Figure 3. The utility of PFS was the most significant influence on the results of the model, with the longest ICER interval ($35,129.27–$66,669.11/QALY). The high expenditure of pemetrexed in the subsequent therapy might prompt the body surface area and cost of pemetrexed to be the high-sensitivity parameters. Osimertinib would become a cost-effective choice when the cost of osimertinib was discounted by 5%, with an ICER of $37,425.45/QALY, lower than the WTP.

**FIGURE 4:**
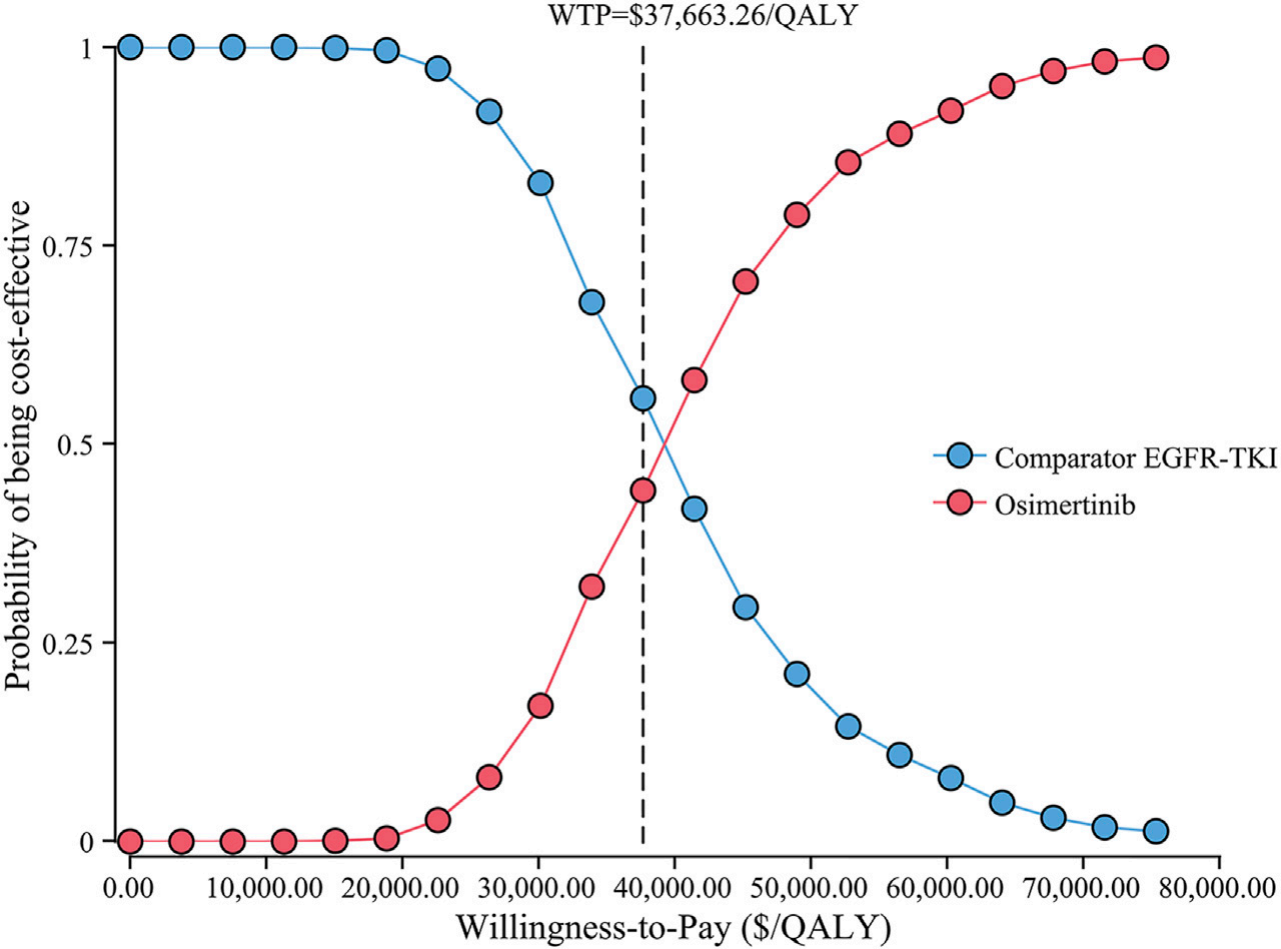
Cost-effectiveness acceptability curve for osimertinib versus the comparator EGFR-TKI. Abbreviations: QALY, quality-adjusted life-year; EGFR-TKI, epidermal growth factor receptor–tyrosine kinase inhibitor; WTP, willingness-to-pay.

**FIGURE 5:**
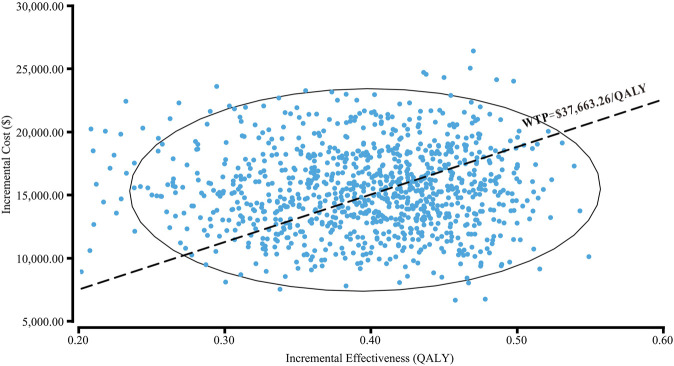
Probabilistic scatter plot of the ICER between osimertinib and the comparator EGFR-TKI. Each dot represents the ICER for one simulation. An ellipse means 95% confidence interval. Dots that are located below the ICER threshold represent cost-effective simulations. Abbreviations: WTP, willingness-to-pay; QALY, quality-adjusted life-year.

The cost-effectiveness acceptability curve showed that osimertinib was cost-effective in approximately 46.4% of the simulations compared with the comparator EGFR-TKI, at the WTP threshold of $37,663.26/QALY. Furthermore, the probability of osimertinib being cost-effective increased from 20 to 80%, when the WTP threshold ranged from $30,000/QALY to $50,000/QALY in China (Figure 4). Compared with the comparator EGFR-TKI, the PSA showed all 1000 iterations fell in the northeast quadrant (Figure 5), and osimertinib added a mean of 0.395 QALY (95% CI; 0.392–0.399) with an additional mean cost of $15,136 (95% CI; $14,924–$15,347), which resulted in a mean ICER of $39,247/QALY (95% CI; $38,549/QALY–$39,946/QALY), similar to the base-case ICER of $39,369.53/QALY.

The subgroup analyses performed by varying the HRs for PFS and OS in race revealed that osimertinib was associated with an ICER of $46,234.58/QALY and $32,352.40/QALY, in the Asian and non-Asian population subgroups, respectively.

## Discussion

With the development and widespread use of new anticancer drugs, the subsequent dramatic increase of the economic burden on the medical healthcare system has become an important topic of concern for policymakers in both developed and developing countries. The challenge of tackling the exorbitant cost of healthcare is becoming an urgent need, especially in a country as populous as China. Over the past decade, EGFR-TKI-targeted therapy had revolutionized the treatment landscape for advanced EGFR-mutated lung cancer patients. However, recent studies have found that the third generation of improved EGFR-TKI can achieve better survival and fewer SAEs due to its selectivity to mutated receptors compared to previous generations (Ramalingam et al., 2020). Osimertinib, the third generation EGFR-TKI, is increasingly being used as a first-line treatment for advanced NSCLC with EGFR mutation and has also been recommended by Chinese Society of Clinical Oncology (CSCO) guidelines in China (Wang et al., 2021). Although the FLAURA study confirmed that osimertinib remarkably extended the median PFS and OS compared with gefitinib or erlotinib, osimertinib was also associated with higher costs. Therefore, from the perspective of pharmacoeconomics, it is significant to evaluate the cost-effectiveness of osimertinib versus first-generation EGFR-TKI as the first-line treatment for previously untreated, EGFR mutation-positive advanced NSCLC.

To the best of our knowledge, this is the first economic assessment study in the Chinese setting using the latest OS data for osimertinib published in January 2020 (Ramalingam et al., 2020). The base-case analysis results demonstrated that osimertinib obtained additional 0.39 QALY and $15,443.78 incremental costs compared with the comparator EGFR-TKI in the first-line treatment of advanced EGFR mutation-positive NSCLC, resulting in an ICER value of $39,369.53/QALY, higher than the WTP threshold ($37,663.26/QALY) of China. Our results suggested that osimertinib might not be a cost-effective option in China. Nevertheless, the ICER is already very close to the WTP of China in our study, indicating appropriate price reduction may make osimertinib cost-effective. Our results are in accordance with economic evaluations in other countries comparing first-line osimertinib, gefitinib, or afatinib in advanced EGFR-mutated NSCLC, which may help clinicians and administrators determine the preferred economic treatment strategy (Aziz et al., 2020; Khoo and Gao, 2021).

In the deterministic sensitivity analysis (DSA), the utility of PFS has the greatest influence on the ICER value with respect to the robustness of the model. In addition, with variations in the utility of PFS, body surface area, cost of pemetrexed, HR for PFS, cost of osimertinib, utility of PD, HR for OS, and discount rate within the specified range, ICERs dramatically increase or decrease, which may induce ICERs lower than the WTP threshold ($37,663.26/QALY), making osimertinib cost-effective. However, the cost of managing serious adverse events has little impact on the model. The PSA illustrated the probability of the cost-effectiveness advantage of osimertinib was 46.4% when WTP was $37,663.26/QALY, suggesting that the first-line treatment of osimertinib for previously untreated advanced NSCLC with EGFR-positive mutation was still not cost-effective as compared to the first-generation EGFR-TKI. As the price of osimertinib decreases, the probability of osimertinib being economically viable increases, consistent with the results of base case and DSA analyses. In China, the ICER value is approaching the WTP threshold, which is economical when the price of osimertinib drops from $28.84/80 mg to $27.40/80 mg. Furthermore, in the non-Asian population subgroup, our study obtained a lower ICER ($32,352.40/QALY vs. $46,234.58/QALY) than the Asian population subgroup, but it did not mean that osimertinib was more cost-effective in the non-Asian subgroup than in the Asian subgroup because the WTP varied in different countries.

There was a FLAURA China study that assessed first-line osimertinib in Chinese patients with EGFRm advanced NSCLC (Cheng et al., 2021). The Chinese cohort showed that the PFS of osimertinib compared with first-generation TKI was 17.8 and 9.8 months, respectively. The median OS of osimertinib and first-generation TKI were 33.1 and 25.7 months, respectively, which were lower than those of the global cohort (Cheng et al., 2021). We calculated the Chinese subgroup data by altering the HRs of PFS (HR = 0.56, 95% CI: 0.37–0.85) and OS (HR = 0.85, 95% CI: 0.56–1.29) in the FLAURA China study. In comparison with the comparator EGFR-TKI, osimertinib obtained an additional 0.34 QALY, with an incremental cost of $16,745.41, resulting in an ICER of $48,798.74/QALY. The ICER of the Chinese cohort showed a higher value than the global cohort, due to the lower survival benefit.

Previously, osimertinib second-line therapy has been used for patients with an acquired EGFR T790M mutation who have progressed on a prior EGFR-TKI therapy (Mok et al., 2017). Moreover, there have been several studies on the economic evaluation of osimertinib as second-line treatment, however, with different conclusions. Wu et al. (2018) reported that treatment with osimertinib did not have a significant economic advantage for patients with advanced EGFR T790M mutation NSCLC after failure of first-line EGFR-TKIs compared with platinum-based chemotherapy in the United States and China, with both ICER values more than $220,000/QALY. On the contrary, other two studies recently published by Bertranou et al. and Guan et al. obtained different results (Bertranou et al., 2018; Guan et al., 2019). The reason might be that the cost of osimertinib was the major driver and varied widely between different studies. On the other hand, individual survival data were diverse based on different clinical trials. Osimertinib as first-line therapy could gain greater health benefit than second-line treatment, especially in patients with identified sensitized EGFR mutations.

At present, evaluation of the economics of osimertinib versus conventional EGFR-TKIs in first-line therapy with EGFR-mutated advanced NSCLC in the United States and Brazil achieved different ICERs per QALY, with $226,527 vs. erlotinib, $231,123 vs. gefitinib, and $219,874 vs. afatinib in the United States (Aguiar et al., 2018). In Brazil, the ICERs per QALY were $162,329, $180,804, and $175,432, respectively (Aguiar et al., 2018). According to the cost-effectiveness threshold criterion of the World Health Organization, these results indicated that osimertinib was also not cost-effective as first-line treatment compared to traditional EGFR-TKIs due to its high cost (Aguiar et al., 2018). Osimertinib was initially marketed in China at the cost of $251.78 per day, much higher than previous EGFR-TKIs, such as gefitinib ($34.92 per day) and erlotinib ($28.88 per day) (Cai et al., 2019). Therefore, two pharmacoeconomic studies on osimertinib versus first-generation EGFR-TKIs were conducted in China based on the primary market price, and both demonstrated that osimertinib was not an economical treatment strategy at that time, with ICERs of $41,512/QALY and $83,766.61/QALY, respectively (Cai et al., 2019; Wu et al., 2019). Excitingly, after the establishment of the National Healthcare Security Administration (NHSA), in October 2018, the indication for osimertinib second-line therapy for T790M mutation-positive advanced NSCLC was successfully entered into the national medical insurance negotiation, and the price was reduced to $73/80 mg. Currently, osimertinib is the only approved first-line treatment EGFR-TKI for advanced or metastatic EGFR-T790M-resistant mutation-positive NSCLC in China. Following the recent negotiations, the market price of osimertinib in China was further decreased to $28.84/80 mg in 2020. Hence, it is necessary to update the economic evaluation results of osimertinib in a timely manner, in order to provide reference for clinicians and decision-makers to formulate reasonable treatment plans.

Compared with the two previous studies published in 2019 in China, our results obtained a lower ICER with $39,369.53/QALY for osimertinib, which were closer to the WTP ($37,663.26/QALY) of China. This was because the high cost of osimertinib was the parameter that had the greatest impact on ICER in previous studies. In addition, the individual survival data on the FLAURA study were not mature then because the median OS results were not released until 2020. It differed greatly from the actual clinical trials when establishing the Markov model only using the original median PFS data on the FLAURA trial. Our findings also suggested that a discount greater than 5% in the osimertinib acquisition cost was required to achieve a cost-effective and accessible alternative, although they had experienced multiple price reductions in China. In addition, considering the unbalanced regional economic development in China, GDP varies greatly between different regions. For example, osimertinib has more than a 50% chance of being cost-effective in the top 11 economically developed areas among 31 provinces of China in 2021, such as Beijing, Shanghai, Jiangsu, Fujian, and Zhejiang, with WTPs of $85,534.88/QALY, $80,837.21/QALY, $63,860.47/QALY, $54,651.16/QALY, and $52,976.74/QALY, respectively (National Bureau of Statistics of China, 2022). As a comparison, osimertinib is distinctly not cost-effective in northwest or southwest China, such as the most underdeveloped provinces Gansu, Heilongjiang, Guangxi, and Guizhou, with WTPs of only $19,023.26/QALY, $21,720.93/QALY, $22,976.74/QALY, and $23,627.91/QALY, respectively (National Bureau of Statistics of China, 2022).

To reinforce the results we obtained, Ezeife et al. (2018) reported that the ICER of osimertinib for previously untreated EGFR-mutant advanced NSCLC in Canada was $223,133/QALY gained, which was above the WTP threshold ($100,000 per QALY), suggesting osimertinib was also not cost-effective at Canadian market price. However, the acceptable cost-effectiveness could be significantly improved when the cost of osimertinib was reduced by 25%, which was consistent with our results and those of Aguiar et al. (2018). Similarly, the ICER values of SG$418,839/QALY and A$432,197/QALY for osimertinib vs. first-generation EGFR-TKIs in first-line therapy with advanced EGFR-mutant NSCLC in Singapore and Australia were obtained, respectively, which was higher than the then WTP (SG$100,000/QALY and A$50,000/QALY, respectively), revealing to be not cost-effective (Aziz et al., 2020; Khoo and Gao, 2021). Another Markov analysis published in Spain indicated a similar cost-effectiveness as compared to first-generation EGFR-TKI because ICER (€273,895.36/QALY) was higher than the commonly accepted threshold €24,000/QALY of Spain while a discount of more than 60% was a cost-effective option (Aguilar-Serra et al., 2019). With the global economic development, the continuous increase of per capita GDP, and the decline of drug prices, osimertinib will become economical in more countries and regions (Ezeife et al., 2018).

There are several limitations to our study. First, survival benefits beyond the monitoring time in the FLAURA trial were evaluated by good-fitting parametric distributions to the publicly available Kaplan–Meier curves, which might cause bias in the model outputs, although the data were validated (Su et al., 2021). Second, the utility values were sourced from published literature because they were not reported in FLAURA. However, the disutility values for SAEs were not considered in our model, which might generate a certain bias to the results. Third, only the most common grade ≥3 SAEs were eligible in our analysis. However, these limitations might be minor factors, according to the one-way sensitivity analysis. Fourth, to simplify the model in our study, only osimertinib or salvage chemotherapy (pemetrexed and cisplatin) would be received as the subsequent therapy, which might not be the same as in the clinical setting, such as radiotherapy and anti-angiogenic therapy. Finally, we modeled proportions of patients receiving subsequent therapy based on the FLAURA trial and guidelines, which might not reflect the current Chinese clinical practice situation precisely. However, due to the new findings in this economic evaluation about osimertinib versus the comparator EGFR-TKI, reflecting the general clinical practice of managing advanced NSCLC, they might be a valuable reference for physicians and policy makers in China.

## Conclusion

In our model, osimertinib first-line treatment for EGFR-mutated advanced NSCLC is an exciting new therapy that leads to a prolonged overall survival and an improvement in QALYs gained, but the ICER is higher than the WTP threshold of China. Therefore, from the perspective of the Chinese healthcare system, osimertinib is unlikely to be considered cost-effective for first-line treatment of advanced NSCLC with EGFR mutations relative to first-generation EGFR-TKI based on its current marketed price. However, a significantly more favorable cost-effectiveness could be achieved when the price of osimertinib was reduced by 5%.

## Data Availability

The original contributions presented in the study are included in the article/Supplementary Material; further inquiries can be directed to the corresponding author.
